# Research in Partnership With Older People—Involvement in Conducting and Analysing Focus Groups

**DOI:** 10.1111/hex.70354

**Published:** 2025-08-20

**Authors:** Nadja Reeck, Anna Völkel, Margarethe Mammes, Dagmar Urbahn‐Schiefer, Bettina Reineking, Tim Stuckenschneider, Anna Levke Brütt

**Affiliations:** ^1^ Department of Health Services Research Carl von Ossietzky University of Oldenburg Oldenburg Germany; ^2^ Faculty of Social Work and Health University of Applied Sciences and Arts Hildesheim Hildesheim Germany; ^3^ Member of the participatory research team Oldenburg Germany; ^4^ Department of Medical Psychology University Medical Center Hamburg‐Eppendorf Hamburg Germany

**Keywords:** focus group, geriatric, older people, participatory research, patient and public involvement, PPI, qualitative research

## Abstract

**Introduction:**

Data collection and analysis in collaboration with co‐researchers has rarely been described, and its impact on the research process and outcomes remains unknown. Thus, this study involved older co‐researchers in the moderation and analysis of focus groups and describes process and outcomes.

**Methods:**

The SeFallED project included collaboration with co‐researchers in the moderation and analysis of focus group interviews. Three focus groups were co‐moderated by an academic researcher and three by a co‐researcher. The co‐analysis was conducted in collaboration with two co‐researchers for three transcripts. Research diaries were maintained throughout the process for reflection. These were supplemented by feedback from the focus group participants. Moderation and analysis were described with regard to the questions and derived categories.

**Results:**

Reflections on the process highlight both facilitators and challenges in involving co‐researchers. A positive atmosphere and training for co‐researchers can be beneficial, whereas the significant time requirements can be challenging. With regard to outcomes, our analysis showed that collaboration led to a greater focus on focus group participants' feelings and facilitated a more profound comprehension of their statements.

**Conclusion:**

The present study provides recommendations for the process of co‐researcher involvement in conducting and analysing focus groups. Involving co‐researchers requires a positive atmosphere, training and adequate time. Furthermore, our findings help to sensitise researchers to the potential impacts on research outcomes.

**Patient or Public Contribution:**

Three co‐researchers collaborated in the moderation and analysis of the focus groups. All three co‐researchers reviewed this paper.

**Clinical Trial Registration:**

The SeFallED project was registered at the German Clinical Trial Register (DRKS00025949, Registration Date: 4 November 2021).

## 1 Introduction

Patient and public involvement (PPI) is increasingly recognised in health research and also acknowledged by research funding agencies [1, 2, 3]. The term PPI stands alongside other terms (e.g., participatory research and patient engagement) that describe partnership research approaches [4]. The National Institute for Health and Care Research (NIHR) describes ‘involvement in research as research being carried out “with“ or “by” members of the public’ [5], emphasising it as an active partnership between the different people that has an influence on research. Amongst others, Charles and DeMaio [6] describe three levels of involvement. The first is called ‘consultation’, where people can give their opinion, but there is no guarantee that it will be considered. In the second level, ‘partnership’, they characterise a collaboration in which researchers and co‐researchers share responsibility regarding planning and decision‐making. The last level is ‘lay control’, where lay researchers have the sole decision‐making power. Previous studies have highlighted the positive and negative consequences of PPI on academic researchers (ARs), co‐researchers and research projects (e.g., improved quality) [7, 8]. Nevertheless, the impact of PPI is highly dependent on contextual factors [9, 10, 11].

Up to now, studies with PPI have often lacked a systematic evaluation [12], and even fewer studies evaluate collaboration in partnership, as characterised by Charles and DeMaio [6], regarding qualitative data collection and analysis. Nonetheless, the review of Jennings et al. [13] describes the analysis of qualitative data in mental health research with PPI as being richer, deeper and enabling alternative understandings. Some initial studies focus on a collaboration in conducting and/or analysing interviews [14, 15, 16, 17, 18, 19, 20, 21, 22, 23, 24, 25]. Most of them conducted individual interviews collaboratively [16, 17, 19, 21, 22, 24, 25], whereas no focus group study could be found. The studies involving co‐researchers in data analysis used different methods (e.g., thematic analysis [19, 20] and inductive content analysis [23]), with involvement often limited to individual analysis phases instead of the entire analysis process [14, 15, 23].

Most of these studies included process reflections by the researchers and co‐researchers, grounded in a documentation of experiences [14, 17, 18, 20, 22, 23, 24], alongside two studies conducting qualitative interviews [15, 21]. Reflections show the need for additional time and organisation [13, 15, 18, 23] and co‐researchers' positive experiences [15, 21, 22, 23]. Some studies describe a more comfortable atmosphere in co‐researcher‐led interviews [16, 21, 22, 23]. Additionally, co‐researcher moderators often brought in their own experiences [25]. Further, Gillard et al. [16] found that co‐researchers focused more on experiences and feelings in their interview questions.

Only a few studies have focused on changes in the research outcomes resulting from collaboration. Two of these studies refer to the reflections of researchers describing awareness of contradictions in interviewees' statements [15] and of the importance of medication [17] as an influence on collaboration. Moreover, Mjøsund et al. [18] analysed conversations between an AR and co‐researchers, and Di Lorito et al. [23] examined differences in interview transcript notes, both reporting better outcomes from collaboration without detailing specific aspects. Three studies investigated differences in themes and frequencies of codes, emphasising a stronger focus on emotions and experiences in co‐researchers' analysis [14, 16, 20]. In summary, these studies reviewed initial experiences of collaboration in qualitative interviews, but evaluations on the impact on research outcomes are scarce.

Although PPI is supposed to increase the quality of research in general [7, 8, 13], it remains unclear which alterations arise from collaborating with co‐researchers in qualitative data collection and analysis. Hence, our study describes how older people collaborated (partnership level [6]) in conducting and analysing focus groups in the ‘Sentinel fall presenting to the emergency department’ (SeFallED) research project in Germany [26]. In an exploratory approach, we examine both the process and the outcomes, placing particular emphasis on the questions posed and the categories derived from the content analysis. We aimed to answer the following research questions:

− How can older people collaborate in conducting and analysing focus groups?
*(reflections of the process: (co‐)researchers' diaries, focus group participants' feedback, questions in focus groups)*
− What is the impact on the outcome when collaborating with older people in focus group analysis? *(impact on outcome: wording/numbers/differentiation of codes)*

## 2 Methods

The *Guidance for Reporting Involvement of Patients and the Public* (GRIPP2) checklist served as an orientation for this article [27] (*see Additional File* *1*).

### 2.1 SeFallED Research Project

The SeFallED project, in which this study is embedded, investigates the functional trajectories and needs of older adults (aged 60 years and older) presenting to the emergency department after a fall. The results serve as a basis for the development of falls prevention interventions [26]. The SeFallED project follows a mixed‐methods approach with four parts: An observational prospective study, a randomised controlled trial (RCT), machine learning approaches and a qualitative study with focus groups [26].

Throughout the research project, a participatory research team (PRT) is involved as consultants. In response to an article in a local newspaper, 56 older individuals with a history of falls expressed interest in participating as members in the PRT ( = co‐researchers). From among them, eight were selected based on the heterogeneity of their socio‐demographic characteristics (e.g., gender, age and education). An expense allowance of €20 per working hour is offered to the PRT members.

The study and PRT involvement were approved by the Medical Ethics Committee of the University of Oldenburg (2021‐120; 2021‐106). Further, the study was registered at the German Clinical Trial Register (DRKS00025949).

### 2.2 Focus Groups

The qualitative part included 12 focus groups to explore older people's perspectives regarding falls prevention measures with the aim of assessing their capabilities, opportunities and motivations after a fall. The interview guide, developed with the PRT, focused on the needs of older adults following a fall, their behaviour regarding falls prevention and the barriers and facilitators influencing engagement in falls prevention interventions. Focus group interviews were audio‐recorded, transcribed verbatim [28] and pseudonymised. The data analysis was oriented around the content‐structuring qualitative content analysis approach of Kuckartz [29]. MAXQDA 2022 software was used by the ARs [30].

**Figure 1:**
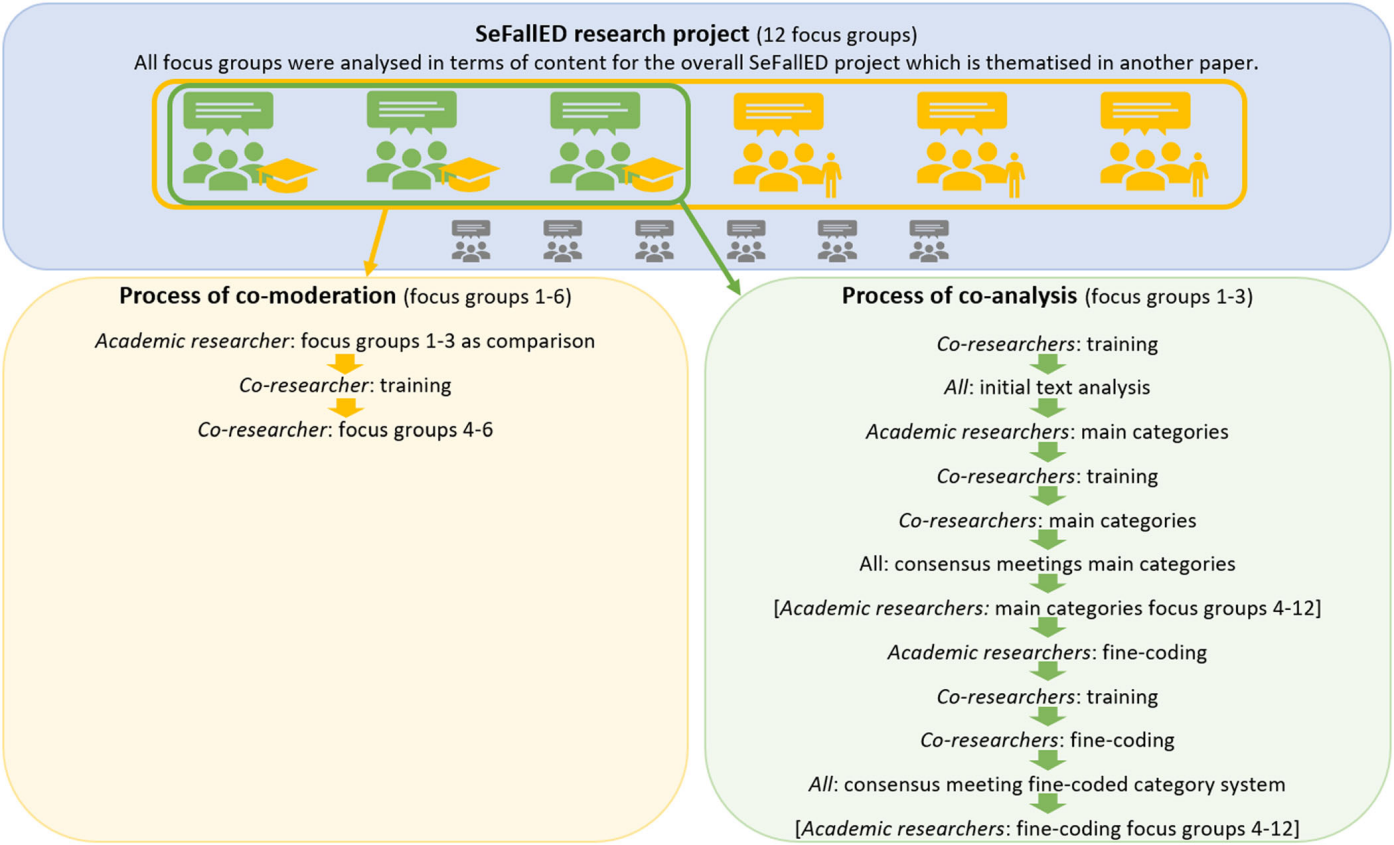
Methodical procedure for the involvement of co‐researchers in the SeFallED focus groups.

Six focus groups (3–6 participants) are considered for the current analysis in this study (see Figure 1). All members of the PRT, which had already been established for approximately 1 year at that time, were asked to voluntarily collaborate in conducting and analysing the focus groups, and three of them were interested (see Table 1 for all involved (co‐)researchers). The role of the co‐researchers was to undertake research tasks in a collaborative partnership. To ensure methodological quality, co‐researchers received training focused on research. In qualitative research, (co‐)researchers collect and analyse data and thereby bring their own subjectivity into the process [31], and our approach ensured that personal affectedness was included as part of this subjectivity.

**Table 1:** Involved (co‐)researchers.

	Data collection	Data analysis	Years in academia	Professional background
***AR1***	Primary moderation in all focus groups	Analysing all transcripts	1	Studied occupational therapist
***AR2***	No involvement	Analysing all transcripts	1	Health services researcher
***AR3***	Co‐moderation focus groups 1–3	Supportive with questions or if no consensus	15	Psychologist
***CR1***	No involvement	Analysing focus groups 1–3	0	Member of the participatory research team; senior citizen, graduate social pedagogue in retirement
***CR2***	No involvement	Analysing focus groups 1–3	0	Member of the participatory research team; senior citizen, social worker and marketing assistant in retirement
***CR3***	Co‐Moderation focus groups 4–6	No involvement	0	Member of the participatory research team; senior citizen, graduate engineer in retirement with experience in teaching, mediation and moderation

The primary moderator was AR1. The co‐moderation of three focus groups was conducted by an AR, while the co‐moderation of the remaining three groups was carried out by a co‐researcher. Our study comprised three focus groups for each co‐moderator, as it was expected that three focus groups would cover 80% of the content [32]. Co‐researcher 3 (CR3) received training in co‐moderation from AR1 to enable a successful partnership [8].

Moreover, the AR‐moderated focus groups were analysed in collaboration with two co‐researchers (see Figure 1). At the beginning, the co‐researchers received a first analysis training by AR1. The initial text analysis was then carried out independently by two ARs and two co‐researchers. After further co‐researcher training, the next analysis step of the deductive coding of the main categories followed [29], in which each tandem pair of ARs and co‐researchers used a deductive main category system based on the Theoretical Domains Framework (TDF) comprising 14 domains that can be assigned to the components' capability, opportunity and motivation [33, 34]. The TDF is characterised by a comprehensive theoretical foundation [33, 34] and provides a framework for co‐researchers. Despite the deductive focus, there was an openness for new inductive main categories. There were five meetings (approximately 29 h in total) with all (co‐)researchers where all coded passages were discussed until consensus was reached. Subsequently, AR1 provided further training for the co‐researchers with a focus on fine‐coding [29], which was conducted by both tandem pairs. Finally, another consensus meeting (approximately 7 h) to agree on a fine‐coded category system was held.

Throughout the entire collaborative process, there was a close and continuous exchange regarding supportive infrastructure, given its importance emphasised in studies focused on older adults [35, 36]. In this context, insights were also drawn from prior experiences with the PRT, which had already established support (e.g., covering taxi expenses and parking facilities).

### 2.3 Analysis of Collaboration

**Figure 2:**
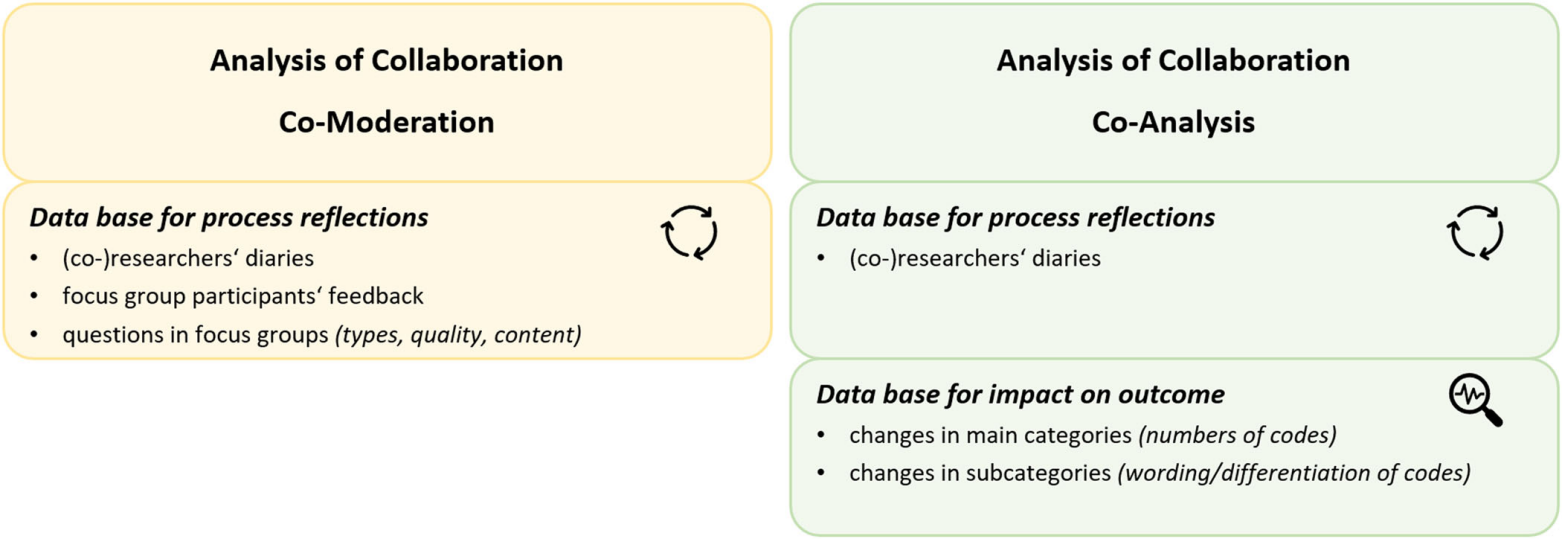
Methodical procedure for the analysis of collaboration in co‐moderation and co‐analysis.

This study follows a mixed‐methods approach to describe the process and outcome of collaboration in conducting and analysing focus groups with older adults. A combination of qualitative (e.g., research diaries) and quantitative (e.g., focus group participants' feedback forms) methods were used for this analysis of the collaboration (see Figure 2).

A positive and trusting relationship, built through previous collaboration in the PRT, provided a solid foundation for co‐researchers to share their views on the collaboration. The collaboration on equal terms also served as a basis for mitigating power imbalances and enabling all those involved to contribute their individual expertise.

It is to mention that all involved co‐researchers were aware of the planned evaluations of their involvement in the focus groups. They were not actively involved in the analysis of the collaboration itself; however, they were involved in reviewing this manuscript.

#### 2.3.1 Co‐Moderation

##### 2.3.1.1 Process Reflections

###### 2.3.1.1.1 (Co‐)Researchers' Diaries

For process reflection, the co‐researcher and the AR answered open‐ended key questions on the atmosphere, their own perception and challenges to encourage reflexion and enable a wide range of possible notes (e.g., ‘How did I feel in the role of (co‐)moderator?’) in a research diary. These reflections took place before and after training, as well as after each focus group. All research diaries were analysed thematically by AR1.

###### 2.3.1.1.2 Focus Group Participants' Feedback

In addition, all focus group participants completed feedback forms where they rated their agreement with 11 statements (e.g., ‘The moderators created a comfortable atmosphere’) on a four‐point Likert scale (1 = do not agree at all; 4 = totally agree) and had the opportunity to make further comments. Statements derived from quality criteria regarding qualitative data collection [37, 38, 39, 40] were discussed with the PRT for finalising the feedback form. AR1 analysed all results of the feedback forms descriptively.

###### 2.3.1.1.3 Questions in Focus Groups

Further, the co‐moderators' contributions were counted and assigned to different types of questions per Helfferich [40]: Narrative‐generating questions, maintenance questions, mirroring back, suggestive questions, immanent steering questions (aspects that have already been named are taken up) and exmanent steering questions (new, yet‐unnamed aspects are introduced). These types of questions enable us to consider differences between the co‐moderators that go beyond the simple count of the number of questions. A colleague (studied Speech and Language Therapist, not involved in the SeFallED project) coded all co‐moderators' contributions to these deductive categories and discussed them with AR1 afterwards.

Additionally, AR1 and her colleague checked the quality of the co‐moderators' contributions following the question rules of Helfferich [40] to investigate whether collaborating with co‐researchers affects quality. These rules note the importance of questions not being ambiguous or difficult to understand. Moderators should use open questions and not multiple questions within a single question. Being judgemental and using specialised terminology should be avoided. Finally, all questions should originate from the interview context and should not trigger feelings of shame or guilt.

As an additional part of the investigation, all questions of the co‐moderators were analysed based on their content. Therefore, the aforementioned colleague inductively coded all questions [29]. She was blinded regarding who asked the question. After coding all questions, she met with AR1 to discuss uncertainties in finalising the category system and the coding.

#### 2.3.2 Co‐Analysis

##### 2.3.2.1 Process Reflections

For process reflection, the data analysis was also accompanied by reflections in the research diaries using key questions by all (co‐)researchers (e.g., ‘How did I find the consensus meeting?’). The use of open‐ended key questions was consistent with the co‐moderation focusing on positive and negative aspects and further suggestions. These reflections were used before and after each training, after every work phase and before and after consensus meetings. AR1 also analysed these research diaries thematically.

##### 2.3.2.2 Impact on Outcome

Further, the changes made in the data analysis as a consequence of the co‐researchers' input were analysed for their impact on research outcomes. This analysis focuses on additional inductive main categories and the number of coded passages in the different main categories after the consensus meetings (numbers of codes). The changes in the subcategory system were also considered, focusing on the wording and the differentiation of the subcategories (wording/differentiation of codes).

## 3 Results

### 3.1 Co‐Moderation

#### 3.1.1 Process Reflections

##### 3.1.1.1 (Co‐)Researchers' Diaries

The training for CR3 in her home environment and focus groups was described as having a pleasant atmosphere. The joint moderation was smooth, and after a short period of familiarisation with the situation, CR3 was satisfied with the course of the interview. In the beginning, AR1 had to face giving up responsibility. Her concerns related to the potential for CR3 to pose an excessive number of questions during the focus group. Table 2 summarises further benefits and challenges. All reflections on the process can be seen in more detail in Additional File 2.

**Table 2:** Process reflections—benefits and challenges.

	*Co‐Moderation*	*Co‐Analysis*
*Benefits*	Good preparation through training with a handout Pleasant, relaxed atmosphere Co‐researcher's behaviour (calm, relaxed)	Well‐structured training with a handout Pleasant, relaxed atmosphere Co‐researchers' insights into different perspectives of focus group participants and into the research process Possibility for co‐researchers to develop their own interpretations Co‐researchers' feeling to be part of the research process and to be heard Co‐researchers' compatibility in working together Co‐researchers' and academic researchers' openness, productive and constructive collaboration New ideas and perspectives for academic researchers, gaining a common understanding after a few meetings Co‐researchers support understanding of focus group participants' statements (e.g., idioms and former job titles)
*Challenges*	Co‐researcher not perceived as peer by focus group participants Academic researcher needed to hand over the reins to a co‐researcher Co‐researcher needed to maintain the interviewer role	Co‐researchers' worries that skills might not be up to the task Co‐researchers' emotional involvement, reminding them of their own fall experiences Integrating work into the daily life of co‐researchers Co‐researchers' limited concentration span Time‐consuming, changes in time schedule Co‐researchers lost the red thread during the coding of subcategories

##### 3.1.1.2 Focus Group Participants' Feedback

Both co‐moderation by the AR and by the co‐researcher showed high levels of satisfaction in the feedback forms (mean values AR3: focus group [FG]1 = 3.97, FG2 = 3.94, FG3 = 3.77; mean values CR3: FG4 = 3.82, FG5 = 3.93, FG6 = 3.94). The free texts were also positive for both (e.g., everything was very good and pleasant atmosphere).

##### 3.1.1.3 Questions in Focus Groups

There was a difference in the number of questions in the focus groups by AR3 and CR3. CR3 spoke up more than twice as often as AR3. There were 41 questions from CR3 (FG4 = 20, FG5 = 5, FG6 = 16) and 13 from AR3 (FG1 = 8, FG2 = 4, FG3 = 1). All questions from the co‐moderators were related to the interview guide.

Considering the co‐moderators' types of questions, differences are recognisable with regard to the steering questions. AR3 used many exmanent steering questions compared to the co‐researcher. The reverse is true for the immanent steering questions (see Table 3).

**Table 3:** Comparison of types of questions from co‐moderators.

Types of questions	Academic researcher*	Co‐Researcher
Maintenance questions	1 of 13 (8%)	5 of 41 (12%)
Mirroring back	4 of 13 (31%)	16 of 41 (39%)
Immanent steering questions	1 of 13 (8%)	7 of 41 (17%)
Exmanent steering questions	6 of 13 (46%)	11 of 41 (27%)
Suggestive questions	1 of 13 (8%)	2 of 41 (5%)

* The data in the table indicates a percentage in excess of 100% for the academic researcher, an occurrence that can be attributed to rounding procedures.

Most questioning rules from Helfferich [40] were well adhered to by both co‐moderators. However, notably both co‐moderators frequently used closed questions (AR3: 77%, CR3: 69%) such as ‘For example, would it have been important for you to have had such an X‐ray beforehand?’ (CR3). Occasionally, they asked multiple questions at once, although this was more the case with AR3 (15% vs. CR3: 6%), as seen in ‘Did the others also see doctors afterwards? So would they also be possible informants?’ (AR3). Judgemental questions such as ‘On the other hand, if you have experienced the treadmill, I always hang (laughing slightly) on the safety harness, it's counterproductive. Right?’ (CR3) were more frequently asked by the co‐researcher (AR3: 8%, CR3: 19%).

With regard to the content of the questions, both co‐moderators focused on service structures (e.g., contact points after a fall) and the focus group participants' fall events. However, AR3 asked many questions about the awareness of support services or related organisational aspects (e.g., costs). Further, her final question solicited further comments from the participants. Conversely, CR3 often focused on everyday habits in her questions, such as the integration of exercise in everyday life. Moreover, CR3 thematised participants' experiences during the training and their motivation for exercising, as well as inquiring about their positions on group offerings and group exercising. Furthermore, she focused on medical care processes (e.g., preferences regarding bureaucratic processes). Another topic addressed by the co‐researcher was joy. Finally, CR3 asked about any general positive or supporting factors that focus group participants experienced after their fall.

### 3.2 Co‐Analysis

#### 3.2.1 Process Reflections

Based on the research diaries, the joint co‐analysis was very challenging for the co‐researchers and time‐consuming (CR2: about 92.5 working hours, CR1: about 113 working hours). However, the friendly relationship between the co‐researchers and the ARs created a positive atmosphere. Moreover, the co‐researchers reported gaining insights into research as very interesting. They also appreciated feeling like they were heard and were able to contribute ideas. The ARs enjoyed exchanging with the co‐researchers to learn more about their perspective. Table 2 summarises further benefits and challenges derived by process reflections. More details about the reflections on this process can be seen in Additional File 2.

#### 3.2.2 Impact on Outcome

Examining the changes in the main categories following the consensus meetings (see Table 4), the most notable observation is the formation of an additional main category, *Health Circumstances*, created on the advice of the co‐researchers.

**Table 4:** Changes in the frequency of main categories after consensus meetings.

Main category	Frequency of academic researchers	Frequency of co‐researchers	Final frequency
Knowledge	13	37	39
Skills	55	19	48
Social/Professional role and Identity	25	11	38
Beliefs about consequences	12	39	38
Reinforcement	27	70	43
Intentions	26	35	27
Memory, attention and decision processes	6	55	35
Environmental context and resources	80	25	280
Social Influences	72	33	86
Emotion	37	47	77
Behavioural regulation	73	31	87
New main category after consensus meetings: Health circumstances			

In general, the collaboration with the co‐researchers led to more coded passages in the transcripts. The coding changed particularly for certain main categories (*Knowledge*, *Beliefs about Consequences*, *Memory, Attention and Decision Processes*, *Environmental Context and Resources*). For example, the ARs coded 13 text passages as *Knowledge*, whereas, in the end, there were 39 text passages coded in this category.

The involvement of the co‐researchers resulted in modifications in the subcategory system. Some changes involved adjustments in wording (e.g., replacing ‘Age group’ with ‘Age’), while others affected the content. For instance, co‐researchers coded a greater number of text passages under ‘Self‐evaluation’ (a subcategory of the main category *Skills*) compared to ARs. Additionally, co‐researchers' involvement led to the subdivision of a certain subcategory used by ARs into several subcategories (e.g., dividing ‘Self‐help’ into ‘Autonomy’ and ‘Expressing Needs’ *(Skills)*). Further, co‐researchers emphasised individual aspects that were included in the ARs' subcategory, resulting in a new subcategory. For example, the text passages of the subcategory ‘Pleasure’ *(Emotion)* were initially only coded as ‘Joy’ *(Emotion)* by the ARs, without their own subcategory. Moreover, in one case, the co‐researchers' involvement resulted in merging the AR's different subcategories into one new overarching subcategory: ‘Attitudes’ instead of ‘Willpower’/‘Acceptance of Help’/‘Willingness to Help’ (*Social/Professional Role and Identity*).

## 4 Discussion

The results section demonstrates the collaboration with co‐researchers in the context of conducting and analysing focus groups. The process reflections highlight the factors that supported the collaboration and the challenges that were encountered, which are addressed in the following discussion. Furthermore, the results show the impact of the collaboration on the research outcome, which is the focus of the second part of the discussion.

### 4.1 Process Reflections

In this study, older co‐researchers were successfully involved in the moderation and analysis of focus groups. However, co‐researchers' diaries also show that this study was challenging, which indicates that a high level of expertise is required to conduct qualitative research. For this reason, based on our experience and the experience of other researchers [13, 23], co‐researcher training is essential even if it cannot replace an academic education. The task of the ARs was to develop the analysis framework and to formulate clear and manageable tasks for the co‐researchers. This was only possible with additional time and organisational efforts to allow for good supervision of the co‐researchers, which has also been emphasised previously [13, 15, 18, 23].

Particularly, co‐analysis led to significant delays in the project schedule, even though a significant amount of time had already been allocated for it (e.g., vacation and illness). Both the AR and the co‐researchers spent much time on co‐analysis, increasing project expenses (205.5 h × €20 = €4110). The entire analysis phase took about 1 year, of which 6 months were needed for the work steps of initiating text work and coding the main categories of three transcripts with the co‐researchers and 2 months for forming the subcategories on the basis of the three transcripts with the co‐researchers. An ethical perspective is also important in this context. Not everyone is willing or able (e.g., lack of time) to participate in research projects with this work intensity, which must be considered when selecting co‐researchers. In addition, regular reflection with those who have agreed to collaborate is important to make any necessary adjustments if the co‐researchers' resources are limited (e.g., changes to the schedule).

In general, all co‐researchers perceived the experience of conducting and analysing focus groups to be positive and expressed interest in insights into the research process. During this process, co‐researchers felt heard and experienced being able to influence the project, which is similar to other studies [15, 21, 22, 23]. In addition, individual co‐researchers reported challenges due to emotional attachment. This was not anticipated by the project team, as the co‐researchers appeared to be less affected by their fall experience. Although emotional triggers are known to be relevant for persons with dementia [23] or life‐limiting illnesses [14], they also apply in the context of frailty. It would be beneficial to discuss emotional triggers with co‐researchers and jointly decide how to address them appropriately (e.g., supervision).

An approachable and trusting atmosphere proved to be an important promoting factor in co‐moderation and co‐analysis, which has also been found in other studies [13, 23]. Similarly, a good relationship between ARs and co‐researchers was found to be beneficial in our study, as in other studies [23, 24]. This was also supported by previous collaboration with the PRT throughout the research project. Despite the fact that the co‐researchers did not act as co‐moderators and co‐analysers in all focus groups due to time constraints, all those involved were able to contribute their expertise, and collaboration took place on an equal footing, as demonstrated by the research diaries. This positive relationship, in conjunction with prior experiences, facilitated the transparent articulation of co‐researchers' requirements for supportive infrastructure. For instance, CR3 communicated her wish to undertake co‐moderation training at home, a request which was subsequently accommodated. Co‐researchers' diaries did not mention any negative aspects regarding infrastructure.

The process of involving the co‐researcher in moderating focus groups was successful, with the co‐researcher contributing numerous questions during the interviews. While she utilised steering questions, she introduced fewer new aspects and primarily focused on elaborating on topics that had already been addressed, in contrast to the AR. It is possible that her own experience provided her with more concrete points of reference regarding the participants' statements, whereas ARs' interactions may be linked to their more theoretical backgrounds. Co‐researcher's questions seemed to approach the participants' everyday life and their feelings, which is supported by Gillard et al. [16].

The feedback forms showed that the focus group participants expressed satisfaction with the interview, irrespective of the co‐moderator. Other studies [16, 21, 22, 23] have found that the involvement of co‐researchers resulted in a more comfortable atmosphere, which was not evident in our study. However, our ratings for the AR were already very high, and so far, the co‐researcher could not have been much higher. Possibly, our evaluation questions were not specific enough. The co‐researcher in our study might not be directly perceived as a peer, as the experience of falling is invisible.

Bengtsson‐Tops and Svensson [21] point out that the loss of a neutral interviewer role can result in interviewees experiencing a sense of insecurity and being influenced by the co‐researcher. Based on our assessment and participants' feedback, this was not an issue in our focus groups. Only at one point, role reversal was evident on the part of the co‐researcher, where she shared her own experiences. Such a role reversal was common in the study of Forbat and Hubbard [25], which might be explained by a lack of specific training, whereas our co‐researcher was trained in the role of a neutral interviewee. To potentially increase the quality of interviews, this should be considered an important part of co‐researcher training.

**Figure 3:**
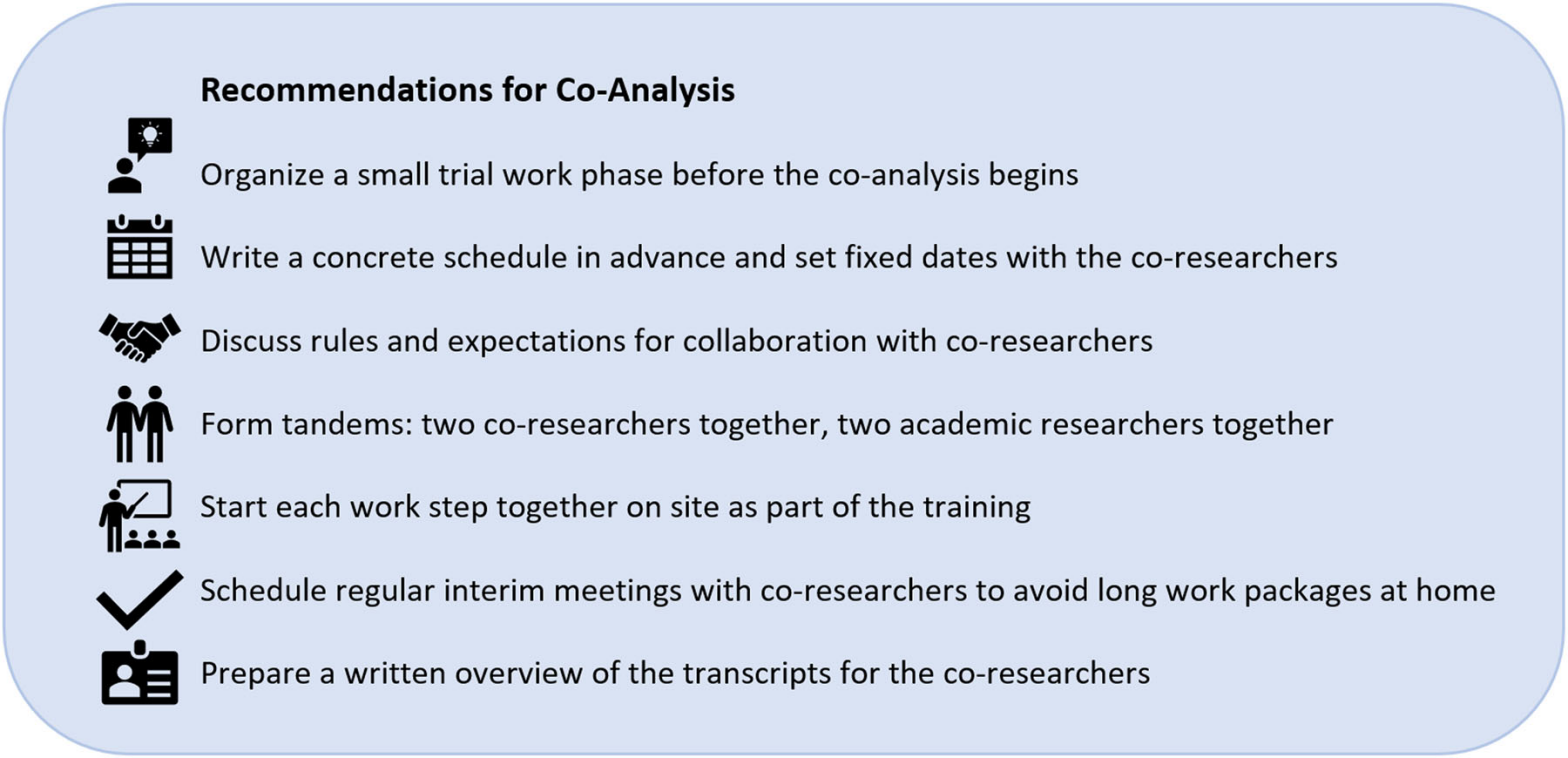
Recommendations for the co‐analysis process based on our experiences.

Co‐analysis on this scale presented a difficult task for the co‐researchers, who needed to be well prepared to avoid being overburdened. Since co‐researchers are usually not very familiar with analysing data, it can be beneficial for them to trial the analysis steps. The utilisation of a deductive category system may also prove advantageous in this context, even if co‐researchers perceived it as quite complex. Consequently, the co‐researchers had already established categories that could serve as a point of reference and also initiate the development of their own main categories. However, they encountered greater challenges in the inductive coding of subcategories. Additionally, it is important to set a concrete timetable together with meetings that are not too long and support them in the planning of work phases at home. Interim meetings should be scheduled during the conduct of an analysis step, so that the co‐researchers can receive feedback on whether they are on the right track. Furthermore, the collaboration between the co‐researchers should be discussed as part of the training programme (e.g., exchange of expectations). Working in tandem was perceived as positive as it enabled an exchange and majorities were avoided. New analysis steps should be trialled directly on site after the theoretical introduction in the training session, so that the co‐researchers can ask questions if necessary. In addition, process reflections indicated that long transcripts could be overwhelming, suggesting that excerpts of transcripts should be created in future projects. Recommendations for organisational adjustments and supporting factors based on our experiences can be seen in Figure 3.

### 4.2 Impact on Outcome

Impacts of collaboration in analysis on research outcome could be determined, as confirmed by Di Lorito et al. [23]. The involvement of co‐researchers resulted in the coding of additional text passages. However, it should be noted that the additional number of codes did not arise solely from the preparatory work of the co‐researchers. Conversely, some additional codes arose from the joint interactions during the consensus meeting (e.g., *Environmental Context and Resources)*. This was also highlighted by Staley and Barron [41], who describe involvement as ‘two‐way learning’ and emphasise that the interaction between ARs and co‐researchers can also lead to changes beyond that of co‐researchers alone, making a difference. This suggests an added value of subsequent joint discussions.

The joint analysis demonstrates that co‐researchers place significant emphasis on emotions, as evidenced by the elevated number of codings allocated to this category. This finding is consistent with the observations reported in several other studies [14, 16, 18, 23]. ‘Autonomy’ and ‘Expressing Needs’ in terms of *Skills* as influencing factors were emphasised more strongly by the co‐researchers. Such a focus on independence is also described in other studies [14, 23]. In our study, the co‐researchers emphasised *Health Circumstances* in creating a new main category, similar to the study of Di Lorito et al. [23]. The findings of Cotterell [14] are also consistent with our results, as our co‐researchers also repeatedly emphasise individuality as an important aspect.

Additionally, looking at the text passages that the ARs considered irrelevant for the main category and thus were not coded, it is notable that removing these might have led to some subcategories not having been created. For example, all coded text passages that later formed the subcategory ‘Age‐related Changes’ were only coded by the co‐researchers. This showed that the co‐researchers focused on issues such as age‐related awareness (‘Age‐related Changes’ and ‘Consequences of Falls’), autonomy (‘Expressing Needs’ and ‘Individual Preferences’) and feelings (‘(Dis‐)Satisfaction’, ‘Pleasure’ and ‘Joy’) in the analysis. These topics serve to reinforce the emphasis on the topics outlined in the preceding paragraph. In summary, the focus of our co‐researchers is strongly connected to the experiences of the participants, including their feelings and needs, as also described in other studies [16, 18, 20].

A special feature was discovered in our study, specifically targeted at a group of older people: the co‐researchers broadened the ARs' perspectives and helped them understand some participant statements previously unclear due to generational differences. The co‐researchers were able to contribute to a better understanding of idioms and expressions, or former job titles that have changed over time. It is conceivable that the presence of co‐researchers, who may exhibit characteristics that diverge more significantly from those of ARs, such as a different age group, could potentially enhance the comprehension of the subject matter. Other factors, including a migration background, may also exert a substantial influence in this regard.

### 4.3 Strengths and Limitations

Studies with older people are often carried out by doctoral students, themselves still quite young [42]. For this reason, the involvement of older people in geriatric research is particularly important for this additional perspective.

A further strength of the study is the collaboration established for the conduction and analysis of qualitative research. Alongside a thorough examination of the process, including an independent researcher for analysing co‐moderator's questions, this study systematically analyses the impact on the research outcome. By using different transcripts for co‐analysis, the effect of co‐analysis was isolated from that of joint moderation.

The collaboration in analysing focus groups is aligned with suggestions from qualitative research to include different perspectives [43]. It should be noted that Jennings et al. [13] and Di Lorito et al. [23] recommend a heterogeneous group of co‐researchers. This should be considered critically with regard to our two co‐researchers involved in analysis, although representing the study's target group, were women of a similar age and professional background. However, more diversity could also pose challenges for consensus and collaboration.

A further limitation of the present study is that it is based on a small number of co‐researchers. There also may have been differences if other ARs instead of co‐researchers had been involved because persons' individuality influences qualitative research [31].

## 5 Conclusion

This study shows how older people can be involved as co‐researchers on a partnership‐based level in conducting and analysing focus group interviews. It therefore extends the work of previous studies that provided initial insights [14, 15, 16, 17, 18, 19, 20, 21, 22, 23, 24, 25] and gives further recommendations for future projects. The process reflections highlight supporting factors and challenges in involving older people. A positive atmosphere and training for co‐researchers fulfilling certain criteria are identified as beneficial, while the significant time requirements posed a challenge.

Furthermore, our study demonstrates the impact of involving co‐researchers on research outcomes. Their involvement can bring participants' feelings and everyday life into focus and enhance understanding of participants' statements. These findings serve to enhance the awareness of researchers with regard to the alterations that ensue from the collaboration with individuals of advanced age. There is a proposal that such collaboration may be beneficial for research projects.

## Author Contributions

**Nadja Reeck:** conceptualisation, writing – original draft, writing – review and editing, formal analysis, investigation, methodology, data curation, validation, visualisation, resources. **Anna Völkel:** writing – review and editing, investigation, validation, formal analysis. **Margarethe Mammes:** writing – review and editing, investigation, validation. **Dagmar Urbahn‐Schiefer:** writing – review and editing, investigation, validation. **Bettina Reineking:** writing – review and editing, investigation, validation. **Tim Stuckenschneider:** writing – review and editing, project administration, data curation, resources. **Anna Levke Brütt:** writing – review and editing, conceptualisation, supervision, funding acquisition, project administration, investigation, methodology, data curation, validation, resources, formal analysis.

## Disclosure

The financial sponsor played no role in the design, execution, analysis and interpretation of data or writing of the study.

## Ethics Statement

The SeFallED project, including involvement of co‐researchers, was performed in line with the principles of the Declaration of Helsinki and was approved by the Medical Ethics Committee of the University of Oldenburg (2021‐120; 2021‐106).

## Consent

SeFallED study participants gave their written consent to be contacted for focus groups. Further, written informed consent for participating in focus groups was obtained from the participants before data collection.

## Conflicts of Interest

The authors declare no conflicts of interest.

## Supporting information

Additional File 1 GRIPP2.

Additional File 2 Process reflections.

## Data Availability

The data that support the findings of this study are available upon request from the corresponding author. The data are not publicly available due to privacy or ethical restrictions. The transcripts of the focus groups and research diaries are not publicly available to maintain the confidentiality and privacy of participants.
