# Supplementary material for: Research in Partnership With Older People—Involvement in Conducting and Analysing Focus Groups
Source: Health Expect. 2025 Aug 20;28(4):e70354. doi: 10.1111/hex.70354 (PMC12367270; doi:10.1111/hex.70354)
Supplement: Supplementary file 1 — Additional File 1 GRIPP2. [file HEX-28-e70354-s001.pdf]

Additional File 1: Guidance for Reporting Involvement of Patients and the Public (GRIPP2) checklist

**Section 1: Abstract of Paper**		
**1a. Aims**	Report the aim of the study relating to PPI	✓
**1b. Methods**	Describe the PPI methods used in the study	✓
**1c. Results**	Report the impacts and outcomes of PPI in the study	✓
**1d. Conclusions**	Summarise the main conclusions relating to PPI in the study	✓
**1e. Keywords**	Include PPI , 'patient and public involvement' or alternative terms as keywords	✓
**Section 2: Background to Paper**		
**2a Definition**	Report the definition of PPI used in the study, including any rationale	✓
**2b. Definition**	Report how your definition links to those provided by other comparable studies	✓
**2c. Concepts and theory development**	Report the way in which PPI is being conceptualised	✓
**2d. Concepts and theory development**	Report any conceptual or theoretical models, or influences, used in the study	✓
**Section 3: Aims of Paper**		
**3. Aims**	Report the aim of PPI in the study	✓
**Section 4: Methods of Paper**		
**4a. Design**	Provide a clear description of methods used for PPI in the study	✓
**4b. Population**	Provide a description of patients, carers and the public involved with the PPI activity in the study	✓
**4c Stages of involvement**	Report on how PPI is used at different stages of the study (e.g. Identifying and prioritising research ideas, designing and managing research, undertaking the research, and dissemination and implementation of research)	✓
**4d. Level or nature of involvement**	Report the level or nature of PPI used at various stages of the study (eg. some people aim for a collaborative approach)	✓
**Section 5: Capture or measurement of PPI impact**		

**5a. Qualitative evidence of impact**	If applicable, report the method used to qualitatively capture the impact of PPI in the study	✓<br>analysing qualitative categories
**5b. Quantitative evidence of impact**	If applicable, report the method used to quantitatively measure or assess the impact of PPI	✓<br>comparison of numbers of coding
**5c. Robustness of measure**	If applicable, report the robustness of the method used to capture or measure the impact of PPI	Not applicable
**Section 6: Economic Assessment**		
**6. Economic Assessment**	If applicable, report the method used for an economic assessment of PPI	Not applicable
**Section 7: Study Results**		
**7a. Outcomes of PPI**	Report the results of PPI in the study, including both positive and negative outcomes	✓<br>e.g. delay in schedule
**7b. Impacts of PPI**	Report the specific impacts that PPI has had on the research, the individuals involved (including patients and researchers), and on communities, including both positive and negative impacts	✓<br>e.g. additional main category, better understanding of statements from focus group participants for the academic researchers, good feeling for co-researchers to be part of the research project
**7c. Context of PPI**	Report the influence of any contextual factors (e.g. availability of funding, policy) that enabled or hindered the impact of PPI	✓<br>e.g. previous collaboration as part of the participatory research team as positive
**7d. Process of PPI**	Report the influence of any process factors (e.g. how users were involved), that enabled or hindered the impact of PPI	✓<br>e.g. too much workload for the co-researchers due to the analysis of three transcripts
**7e. Concept and theory development**	i) Report any key conceptual or theoretical developments in PPI that have emerged from the study	✓<br>recommendations for future projects, reported impact on research outcome
	ii) If applicable, report any testing of conceptual or theoretical models	Not applicable
**7f Measurement**	If applicable, report all aspects of instrument development and testing (e.g. validity, reliability, feasibility, acceptability, responsiveness, interpretability, appropriateness, precision)	Not applicable
**7g. Economic assessment**	Report any information on the economic cost or benefit of PPI	✓<br>e.g. working hours of co-researchers and compensation for expenses
**Section 8: Discussion and Conclusions**		

**8a. Outcomes**	Comment on the extent to which PPI influenced the study overall. Describe positive and negative effects	✓
**8b. Impacts**	Comment on the different impacts identified in this study and how they contribute to the new knowledge	✓
**8c. Definition**	Comment on the definition of PPI used in the study and whether you would suggest any changes	Not applicable
**8d. Conceptual development and theory development**	Comment on how this study adds to conceptual or theoretical development of PPI	✓
**8e. Context and process**	Comment on how context and process factors influenced PPI in the study	✓
**8f. Measurement and capture of PPI impact**	If applicable, comment on how well PPI impact was captured or measured in the study	✓ See Strengths and Limitations
**8g. Economic information**	If applicable, discuss any aspects of economic cost or benefit of PPI, particularly any suggestions for future economic modelling.	Not applicable
**8h. Critical perspective**	Comment critically on the study, reflecting on the things that went well and those that did not, so others can learn from this experience	✓

Staniszewska S, Brett J, Simera I, Seers K, Mockford C, Goodlad S, Altman DG, Moher D, Barber R, Denegri S *et al*: **GRIPP2 reporting checklists: tools to improve reporting of patient and public involvement in research**. *Research Involvement and Engagement* 2017, **3**(1):13.
