# Supplementary material for: Research in Partnership With Older People—Involvement in Conducting and Analysing Focus Groups
Source: Health Expect. 2025 Aug 20;28(4):e70354. doi: 10.1111/hex.70354 (PMC12367270; doi:10.1111/hex.70354)
Supplement: Supplementary file 2 — Additional File 2 Process reflections. [file HEX-28-e70354-s002.pdf]

Additional File 2: Process reflections based on research diaries

Co-moderation		
**Work step**	**Co-researchers‘ reflections**	**Academic researchers‘ reflections**
Training	• *handout made it possible to read everything again at home*<br>• *optimistic attitude*<br>• *AR1's approachable and friendly nature contributed to good atmosphere*<br>• *felt good prepared*	• *training in co-researcher's home environment contributed to a pleasant and relaxed atmosphere*
Focus groups	• *pleasant and relaxed atmosphere*<br>• *no problems while conducting the three focus groups*<br>• *no need for further coordination during interviews, it all happened automatically*<br>• *at the first joint focus group initially cautious (first had to see how and which in-depth questions she could ask without disturbing the main moderator)*<br>• *reinforced the participants' statements or summarised statements in order to trigger new impulses in all focus groups → would have liked to have done this more, but avoided doing so in order not to diminish the openness (even without these impulses, the discussion usually went in the same direction)*<br>• *aim: carry out aspects that are as tangible as possible (e.g. taster day)*	• *pleasant and relaxed atmosphere*<br>• *CR3's behaviour calm and relaxed*<br>• *participants did not perceive CR3 as peer, more as professional employee*<br>• *unproblematic*<br>• *no further guidance or consultation during the focus groups*<br>• *in the beginning of the first joint focus group: for a short moment AR1 thought it might be too many questions asked by CR3 → little difficult to hand over the reins to CR3 as a co-researcher with no research experience*<br>• *CR3 sometimes also contributed her own ideas and thoughts and thus did not maintain the neutral interviewer role → no problem from AR1's point of view, as it did not interfere with or significantly influence the course of the interview*
**Co-analysis**		
**Work step**	**Co-researchers‘ reflections**	**Academic researchers‘ reflections**
First training	• *no experiences in analysing interviews or focus groups*<br>• *looked forward for the analysis and were curious about new insights*<br>• *CR2: some worries that skills might not be up to task → based on previous positive experiences, she was convinced that everything would work out well*<br>• *enjoyed first training*<br>• *well structured and work assignments clearly formulated*<br>• *handout supportive*<br>• *atmosphere positive, relaxed casual conversation*<br>• *felt well prepared*	• *handout helpful as training guide*<br>• *positive atmosphere*<br>• *casual, relaxed conversation in which all questions were answered*
Initiating text work	*CR1*<br>• *liked this work phase and enjoyed working on texts with a specific goal in mind*	*Not involved*

	• *really immerse herself in the focus groups and the research topic*<br>• *liked to come up with own interpretations*<br>• *positive feeling to be part of a research process*<br>• *themes stayed with her for a long time*<br>• *enough time for this task as a supporting factor*<br>• *working documents helpful as little reminder*<br>• *reading the first focus group, her emotions became too dominant → felt extremely emotionally involved and reminded of her fall (longer break necessary)*<br>• *problems in integrating the work in her daily life*<br>• *difficulties due to a limited concentration span*<br>• *took more time as expected, but always a pleasure*<br>*CR2*<br>• *very strenuous (e.g. looking back to see who was which person)*<br>• *workload too high, also due to other tasks as part of the regular tasks of the PRT*<br>• *difficulties due to a limited concentration span*<br>• *inner need to finish the task as quickly as possible*<br>• *insight into the different perspectives very interesting*	
Second training	*CR1*<br>• *looking forward to second training session*<br>• *liked the training, eased her fears about the next work phase*<br>• *good atmosphere (e.g. feeling no question was stupid)*<br>*CR2*<br>• *no expectations, open to it*<br>• *training confused her, she became restless*<br>• *difficulty in concentrating*<br>• *did not feel well prepared → wanted to go home first and did not want to ask further questions or start the new work step on site at the university as planned*<br>• *atmosphere positive, emphasising AR1's friendliness and composure → supportive despite everything*	• *relaxed conversation*<br>• *co-researchers interested*<br>• *starting the work phase on site was rejected by the co-researchers (also suggestions such as taking a break)→ would have been very helpful, as questions often only arise when starting a task*
Main categories	• *in the beginning they first had to exchange ideas about their working methods → approaches and demands were exchanged to reach a common denominator*<br>*CR1*	*Not involved*

	• *documents provided a good thread for work phase*<br>• *sometimes no longer able to distinguish the main categories from one another*<br>• *good feeling that the analysis became easier for her over time → sense of success*<br>• *at the end of this work phase, increasingly confused about the categorization*<br>• *difficult to maintain focus and not get lost in the individual passages*<br>• *collaboration with CR2 very positive*<br>*CR2*<br>• *uncertainty continued*<br>• *high level of inner stress ('perfection stress')*<br>• *task very difficult and complex → problems understanding main categories and applying them to focus groups and research questions*<br>*Both*<br>• *stressful to have to go through all the transcripts again*<br>• *interest in completing the work phase quickly*<br>• *their own personal experiences came up again (distracting, challenging)*	
Consensus meetings main categories	*CR1*<br>• *positive mood before first consensus meeting*<br>*CR2*<br>• *looking forward to reunion*<br>• *hoped for more understanding for the analysis*<br>• *hoped that everything would be finalised in the first consensus meeting*<br>*Both*<br>• *enjoyed meetings, good atmosphere and intensive, in-depth discussions*<br>• *felt heard, positive that they could convince academic researchers of ideas*<br>• *good results from consensus meetings*<br>• *length of the consensus meetings negative*<br>• *providing snacks and drinks positive*<br>• *underestimated amount of work (five consensus meetings)*<br>• *difficult to maintain motivation*	• *were looking forward to the exchange and getting to know new perspectives*<br>• *enjoyed the consensus meetings, good atmosphere, very productive and constructive*<br>• *new ideas and perspectives became visible (e.g. academic researchers have sometimes not seen the connection to the fall topic)*<br>• *From the third consensus meeting, feeling that a common understanding had been established, knew how co-researchers think*<br>• *discussions not tough and consensus could always be found*<br>• *Expectation of time-consuming step was fulfilled, work step took even more time than expected*<br>• *stressful, time schedule had continued to shift → co-researchers limited in terms of time due to their everyday and leisure activities (e.g. vacations) → another necessary appointment for a consensus meeting could quickly mean a further delay of two weeks*
Third training	• *curious about this analysis step and positive about it*<br>• *expected interesting results*	• *satisfied*

	• *felt well prepared, no open questions*<br>• *handout positive*	• *motivation of the co-researchers positive, despite the time-consuming analysis steps so far*
Fine coding	• *did not like the work phase*<br>• *no motivation*<br>• *difficulties with task → often had to read in transcripts for understanding statements, sometimes not able to understand assignment of codes*<br>• *felt like everything was repeating itself, confused*<br>• *time required too high*	*Not involved*
Consensus meeting fine coding	• *were looking forward to see each other again*<br>• *hoped it will not take too much time*<br>• *positive atmosphere*<br>• *happy that the fine coding was finished after one meeting*<br>• *content of the consensus meeting good*<br>• *pleased that agreement was reached so well*<br>• *gained more clarity about results → put everything together in meaningful way*	• *looking forward to the joint meeting*<br>• *concerned that it might take too much time*<br>• *worried in advance that motivation of co-researchers might be low and there would be no discussion and that everything would simply be quickly dismissed*<br>• *good atmosphere*<br>• *relieved that they finished all in one consensus meeting*<br>• *feeling that co-researchers wanted to finish quickly and there was slightly less discussion than at previous meetings → but co-researchers still contributed ideas and discussed individual aspects and did not just approve everything*
